# Acupuncture for abdominal wall endometriosis: A case report

**DOI:** 10.1097/MD.0000000000036572

**Published:** 2023-12-15

**Authors:** Xiufan Du, Jiahao Tang, Lixia Zhang, Wei Yi

**Affiliations:** a Clinical Medical School of Acupuncture, Moxibustion and Rehabilitation, Guangzhou University of Chinese Medicine, Guangzhou, Guangdong, China.

**Keywords:** acupuncture, case report, endometriosis, infertility

## Abstract

**Introduction:**

Endometriosis refers to a series of symptoms caused by the presence of endometrial-like tissue outside the uterine cavity. In extrapelvic endometriosis, abdominal wall endometriosis (AWE) is very common. Acupuncture therapy has been widely used as an alternative therapy to treat multiple diseases, such as sequelae of stroke, pain, and facial paralysis. To our knowledge, case reports of acupuncture for the treatment of AWE has not been reported. We report a case of acupuncture in the treatment of abdominal endometriosis.

**Rationale:**

AWE could result in symptoms including pelvic pain, dysmenorrhea, and infertility. Acupuncture might be effective in the treatment of the disease.

**Patient concerns:**

A 38-year-old woman complained of the aggregation of pain in a mass, which is located in her abdominal wall.

**Diagnoses:**

The patient was diagnosed with AWE, surgical history (excision of deep abdominal wall mass, repair of abdominal wall defect with patch). According to traditional Chinese medicine theory, traditional Chinese medicine diagnosis is Zhengjia (qi stagnation and blood stasis pattern).

**Interventions:**

Combined with the theory of disentanglement, we use acupuncture, cupping, and needle therapy to promote qi circulation, activate blood circulation, relieve pain, and dissipate masses.

**Outcomes:**

After treatment, abdominal ultrasound showed that the mass gradually decreased.

**Conclusion:**

Acupuncture can effectively relieve the pain caused by abdominal endometriosis and reduce the size of abdominal endometriosis masses.

## 1. Introduction

Endometriosis refers to a series of symptoms caused by the presence of endometrial-like tissue outside the uterine cavity, including glands and stroma. The ectopic endometrium continues to grow, infiltrate, and bleed under the influence of hormones, leading to the formation of nodules and masses, resulting in symptoms such as pelvic pain, dysmenorrhea, and infertility. In extrapelvic endometriosis, abdominal wall endometriosis (AWE) is very common.

Modern medicine primarily relies on hormone therapy and surgery for treatment ^[1,2]^; however, available drugs are not targeted therapy, and they are only effective in treating endometriosis-related pain.^[3]^ In addition, both approaches come with varying degrees of adverse reactions and the possibility of recurrence.^[4]^ In clinical practice, acupuncture has been used to alleviate pain, regulate menstruation, and reduce the size of masses in the treatment of abdominal endometriosis. It offers advantages such as a low recurrence rate and minimal adverse reactions. This article reports a case study of acupuncture treatment for abdominal endometriosis, aiming to provide a potential alternative for the clinical management of abdominal endometriosis and inspire further exploration of acupuncture as a treatment option for abdominal endometriosis.

## 2. Case presentation

### 2.1. Clinical presentation

**Figure 1.**
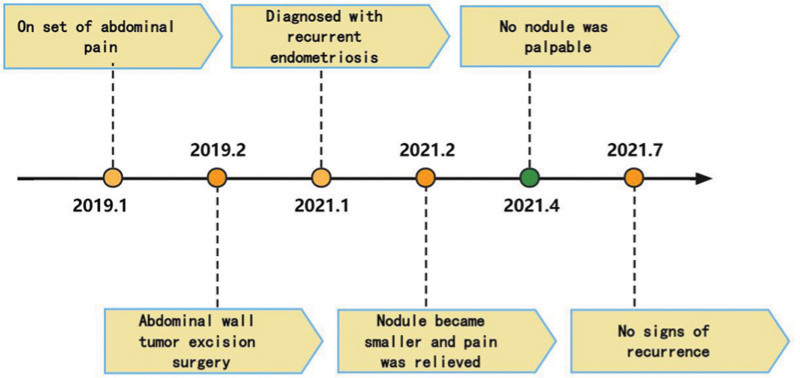
Timeline of this case report.

**Figure 2.**
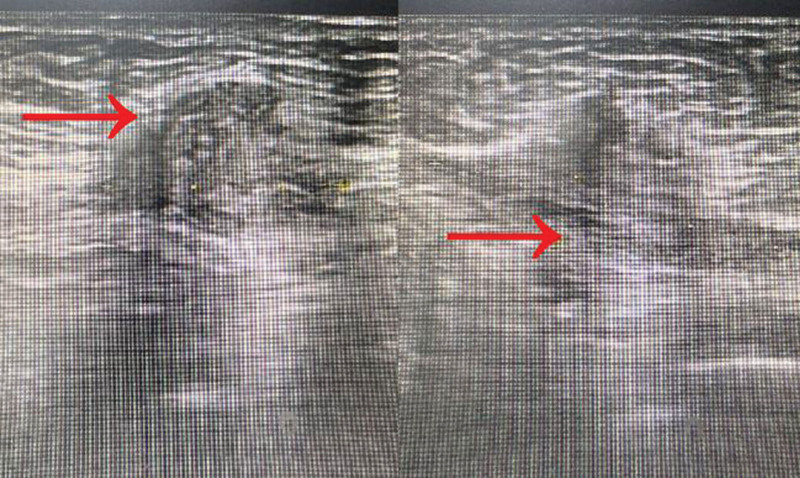
Preoperative ultrasound of abdominal wall masses showed that hypoechoic mass was found in the soft tissue of the abdominal wall, ranging from 18 mm × 41 mm × 27 mm, with irregular shape, clear boundary, and uneven internal echo.

**Figure 3.**
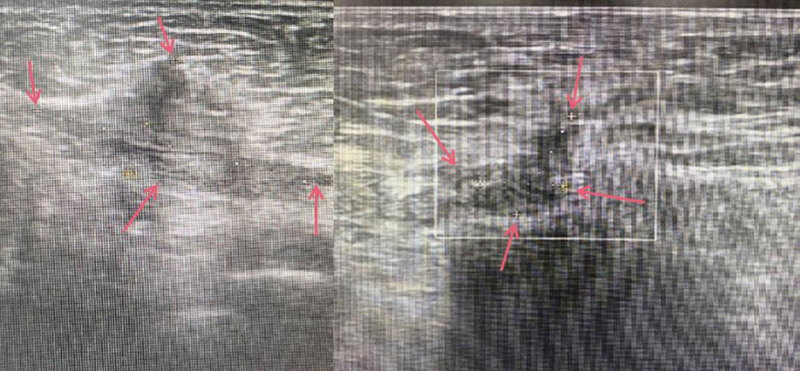
The irregular low echo area was found in the deep muscle layer behind the abdominal wall incision, the range was about 12 mm × 9 mm, the boundary was unclear, and the internal echo was uneven.

**Figure 4.**
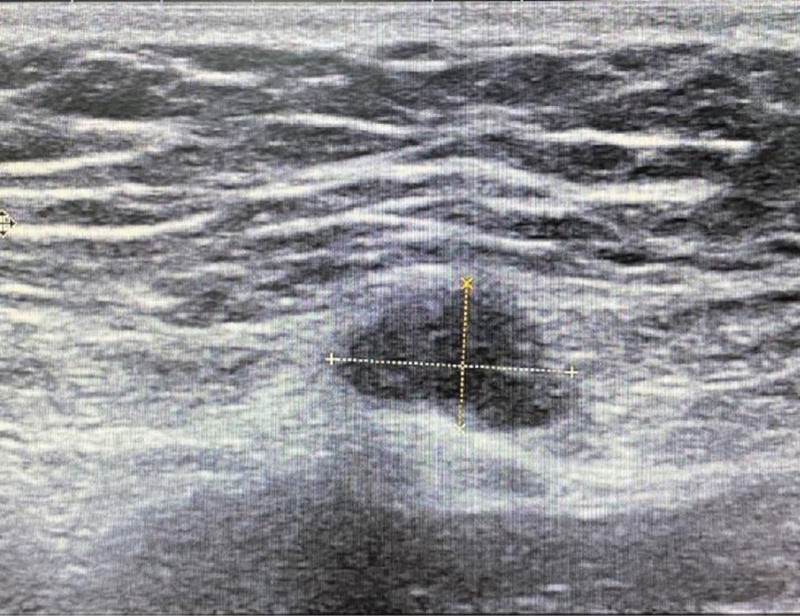
Ultrasound of abdominal wall masses showed a low echo mass in the lower left quadrant area where the surgery scar lied. Its size was about 33 mm × 7 mm × 14 mm, 17.5 mm away from the surface of the abdomen with uneven internal echoes, and the margin was clear.

A 38-year-old lady complained of the aggregation of pain in a mass, which is located in her abdominal wall (Fig. 1). It lasted for a month. She found a mass under her belly button in September, 2017, but she did not give it much of attention. Around January, 2019, she paid a visit to The First Affiliated Hospital of Guangzhou University of Traditional Chinese Medicine after she felt the hardness of the mass in her abdominal is progressing and there was notable pain when she pressed it. Preoperative ultrasound was performed at the hospital (Fig. 2). Then she received abdominal wall tumor excision surgery and patch repair for abdominal wall defect after examinations and assessment. The biopsy of the tumor suggested endometriosis in abdomen. Patient recovered well after surgery and came back for follow-up examination. Color Doppler ultrasound of abdominal wall showed that there was a low echo area measuring 12 mm × 9 mm (square-like) within the muscular layer whose margin was clear (Fig. 3). Given the clinical symptoms, it was considered to be a patch in the abdominal wall. The patient spotted a coin-size nodule around 3 cm lower left to her belly button that has been gaining its size progressively since a month ago. A day before, the patient underwent color Doppler ultrasound that suggested a low echo mass in the lower left quadrant area where the surgery scar lied. Its size was about 33 mm × 7 mm × 14 mm, 17.5 mm away from the surface of the abdomen with uneven internal echoes, and the margin was clear (Fig. 4). A highly probable diagnosis was recurrent endometriosis after laparoscopic resection surgery. Now the patient is presented to the outpatient department. Patient’s medical history includes previous uterine fibroids and breast hyperplasia. Since a cesarean section in 2011, the patient has been experiencing dysmenorrhea until now. Obstetric history: married, gravida 1, para 1, abortus 0. Current symptoms: slightly tired mentally, occasional breast distension and pain, easily irritable and restless, occasional chest tightness and discomfort, dry and bitter mouth, occasional stabbing pain below the umbilicus, aggravated pain when menstruating or applying abdominal pressure, no obvious tenderness, but has palpation pain. Last menstrual period: January 8, 2021, lasted for 7 days, regular cycle, with dysmenorrhea, heavy flow, bright red color with blood clots, poor appetite, disturbed sleep, normal bowel movements, dark tongue coating with yellow and greasy appearance, and wiry and tight pulse.

### 2.2. Interventional procedure

According to traditional Chinese medicine (TCM) theory, we determined that the western medicine diagnosis is AWE with surgical history (excision of deep abdominal wall mass, repair of abdominal wall defect with patch). TCM diagnosis: Zhengjia (qi stagnation and blood stasis pattern). Treatment principles: promote qi circulation, activate blood circulation, relieve pain, and dissipate masses. Treatment method: acupuncture combined with resolving masses therapy. First, locate and puncture the superficial and deep collaterals in the abdominal wall nodules or lumbosacral region. In this patient, there is significant blood stasis in the lumbosacral region. Using a No. 7 injection needle, puncture the superficial collaterals 3 to 5 times, followed by applying a No. 4 cupping jar for bloodletting. The amount of bloodletting is approximately 5 mL, leaving the cupping jar in place for 5 minutes. After removing the jar, wipe the puncture site with a sterile cotton ball. For the abdominal mass, locate its position on the body surface, avoiding the patient’s abdominal surgical scars. Apply mild moxibustion therapy using a 0.30-mm × 40-mm acupuncture needle. Heat the needle with an alcohol lamp until it turns red and bright, then quickly pierce the skin to a depth of approximately 20 mm without manipulation. Leave the needle in place for 3 minutes. Then, obliquely insert a 0.30-mm × 25-mm fine needle at the Shenting (DU24) and Baihui (DU20) points to a depth of 10 mm. For the bilateral Shuimeng (DU26), Tian Shu (ST25), Guan Yuan (CV4), bilateral Daheng (SP15), left Sanyinjiao (SP6), bilateral Yinlingquan (SP9), and left Shangqiu (SP5) points, use a 0.30-mm × 40-mm needle to insert directly to a depth of 10 to 30 mm. After needle insertion, apply even reinforcing and reducing manipulation and retain the needle for 20 minutes once qi is obtained. Comprehensive treatment is conducted twice a week, with a 3- to 4-day interval between each treatment. A total of 8 treatments constitute 1 course of treatment, wherein the cupping therapy and needle insertion are performed only once a week.

### 2.3. Follow-up and patient’s perspective

**Figure 5.**
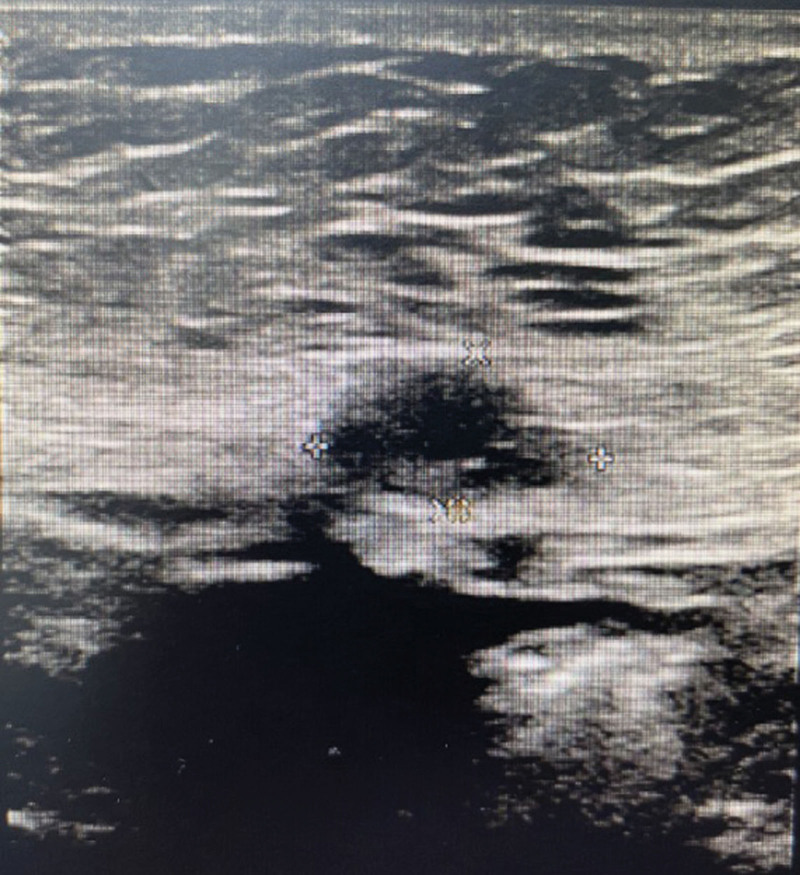
Ultrasound of abdominal wall masses showed a hypoechoic mass within the muscle layer of the lower left abdomen, measuring approximately 22 mm × 4 mm × 10 mm, located 16.6 mm from the body surface.

On February 17, 2021 (after the first course of treatment), the patient returned for a follow-up visit. The patient reported that the nodule below the umbilicus had become smaller and there was no obvious pain. Menstrual pain had significantly improved compared to before, and there was an improvement in irritable mood. On February 16, 2021, a repeat color Doppler ultrasound showed a hypoechoic mass within the muscle layer of the lower left abdomen, measuring approximately 22 mm × 4 mm × 10 mm, located 16.6 mm from the body surface (Fig. 5). The patient was advised to continue treatment with the previous method.

On April 14, 2021 (after the third course of treatment), the patient returned for a follow-up visit. The patient reported that no nodules were palpable below the umbilicus, and menstrual pain was painless. The patient had stable emotions and good sleep. The tongue was pale with a thin yellow coating, and the pulse was weak and wiry. Physical examination revealed no nodules or tenderness in the lower abdomen. Sparrow-pecking moxibustion was applied to the affected area for 10 to 15 minutes, and the remaining treatment method remained the same. The frequency of visits was reduced to once a week, and consolidative treatment was continued for an additional 8 sessions. Four sessions constituted 1 course of treatment, and a total of 2 courses of treatment were completed.

**Figure 6.**
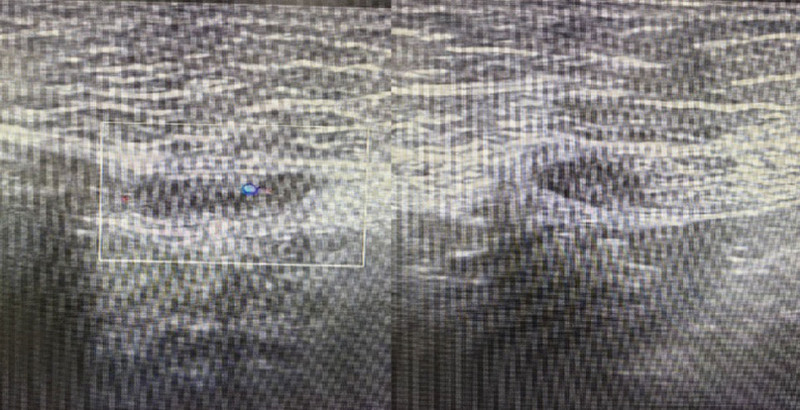
Ultrasound of abdominal wall showed a hypoechoic mass within the muscle layer of the lower left abdomen, measuring approximately 12 mm × 8 mm.

On July 28, 2021 (after 2 courses of consolidative treatment), the patient returned for a follow-up visit. The patient reported similar symptoms, with a generally good condition. No nodules were palpable below the umbilicus, and there was no tenderness. On July 25, 2021, a repeat color Doppler ultrasound showed a hypoechoic mass within the muscle layer of the lower left abdomen, measuring approximately 12 mm × 8 mm (Fig. 6), which was similar in size to the abdominal wall patch seen on the ultrasound after the surgery in 2019. Up to the present follow-up, the patient reported no recurrence of the nodules.

## 3. Outcome

After 8 courses of treatment, the size of abdominal mass changed from 18 mm × 41 mm × 27 mm to 12 mm × 8mm, which was consistent with the size of abdominal wall patch. Abdominal examination did not touch the mass, abdominal tenderness. Our patient’s menstruation was completely normal and irritability improved.

## 4. Discussion

The etiology of AWE is complex and multifactorial, and its exact causes and mechanisms are not yet fully understood. The main theories include the implantation theory (retrograde menstruation and iatrogenic implantation), metaplastic theory of coelomic epithelium, hematogenous and lymphatic dissemination theory, and immunological theory.^[5,6]^ The lesions of this condition are located between the peritoneal layer and the skin, usually occurring at or near surgical scars. The main symptoms are cyclic pain and tender nodules in the abdominal scar area. With the increasing prevalence of abdominal surgeries, especially cesarean sections, the incidence of AWE has been rising.^[7]^ Surgery is currently the only effective treatment for AWE. However, the risk of recurrence should not be ignored, as there may be incomplete removal of lesions, which is one of the major reasons for recurrence. Additionally, iatrogenic implantation during surgery can also contribute to recurrence. Acupuncture, as an effective treatment option for menstrual disorders, ovarian insufficiency, and infertility, is widely accepted in China and around the world.^[8,9]^ However, there are few reports on its role in the treatment of AWE. This article reports a case of acupuncture treatment for AWE, aiming to provide a potential alternative for the clinical management of this condition and inspire further exploration of acupuncture in the treatment of AWE.

Based on the symptoms and characteristics of AWE, it can be classified within the scope of “Zhengjia” in TCM. The pathological key lies in blood stasis, which is related to pathogenic factors such as qi stagnation, damp-heat, kidney deficiency, qi deficiency, and cold congelation. “Zhengjia” represents the accumulation and blockage in the uterus and its related meridians. The occurrence of this disease is often the result of a combination of underlying deficiency and external factors such as the 6 climatic evils, the 7 emotions, or traumatic invasion, which obstruct the circulation of qi in the organs or meridians, causing the condensation of blood stasis, cold pathogen, or phlegm-dampness. Various pathogenic factors often interact with each other, gathering and accumulating in the meridians and vessels related to the uterus, gradually transforming into “Zhengjia.” In this case, the patient had a history of cesarean section, and dysmenorrhea began after childbirth, suggesting damage to the meridians during the cesarean section, leading to blood stasis and accumulation in the abdomen. This caused pain due to stagnation. Although the surgical removal of the deep abdominal mass resolved the blood stasis, the underlying blood stasis mechanism was not corrected, and the damaged meridians did not recover, resulting in the gradual reformation of blood stasis in the affected area and recurrence of endometriosis. Combining the tongue and pulse diagnosis, it indicated a pattern of qi stagnation and blood stasis. Acupuncture treatment was applied to resolve the stagnation and dispersion of blood stasis, promote the circulation of qi, and provide warmth and pain relief. Initially, fire needling was used at the nodules to disperse the stagnation and provide warming and pain-relieving effects. Once the nodules showed signs of dissipation, a gentler sparrow-pecking moxibustion was used to warm and promote the flow without damaging the body fluids. Cupping therapy was used on the blood vessels in the lumbar-sacral region to eliminate stasis and facilitate the flow of qi and blood. The patient’s emotional and mental state is not smooth. To regulate the spirit, points such as Shenting (GV 24) and Baihui (GV 20) are selected. Considering the patient’s long-standing illness and deficiency, points along the spleen and stomach meridians are chosen, including Huaroumen (ST 24), Tianshu (ST 25), Daju (ST 27), Sanyinjiao (SP 6), Yinlingquan (SP 9), and Shangqiu (SP 5), to nourish the postnatal essence and strengthen the foundation. Guanyuan (CV 4) of the Ren meridian is selected to supplement the original qi, consolidate the prenatal qi, regulate the Chong and Ren channels, and simultaneously alleviate abdominal pain symptoms. The combination of these techniques aims to warm and disperse stagnation, promote the circulation of qi and transform stasis, and warm the meridians to alleviate pain.

### 4.1. Limitation

Due to the small number of cases, which may lead to biased outcomes and questions about reproducibility, we look forward to the opportunity to conduct more detailed controlled clinical studies.

## 5. Conclusions

This article reports a case of successful acupuncture treatment for AWE, which resulted in the reduction of the mass and relief of pain. Our report suggests that alternative therapy such as acupuncture can effectively alleviate the symptoms of AWE. However, further standardized clinical studies are still needed to validate the effectiveness and safety of this treatment.

## Author contributions

**Investigation:** Xiufan Du, Jiahao Tang, Lixia Zhang.

**Resources:** Xiufan Du, Wei Yi.

**Writing – original draft:** Xiufan Du, Jiahao Tang.

**Writing – review & editing:** Xiufan Du, Jiahao Tang, Wei Yi.

**Supervision:** Wei Yi.

**Validation:** Wei Yi.
